# Nodular Fasciitis of the Auricle: A Case Report

**Published:** 2013

**Authors:** Mohammad Reza Majidi, Amir Hossein Jafarian, Shirin Irani

**Affiliations:** 1 *Sinus and Surgical Endoscopic Research Center, Mashhad University of Medical Sciences, Mashhad, Iran.*; 2 *Department of pathology, Quaem Hospital, Mashhad University of Medical Science, Mashhad, Iran.*; 3 *Departments of Otorhinolaryngolog, Quaem Educational Hospital, Mashhad University of Medical Sciences, Mashhad, Iran*.

**Keywords:** Ear, Ear Auricle, Excision, Nodular Fasciitis

## Abstract

**Introduction:**

Nodular fasciitis is described as a benign reactive proliferation of myofibroblasts. Due to its rapid-growing nature, a precise clinical diagnosis is difficult and the condition is frequently misdiagnosed as malignant lesions.

**Case Report:**

In this study, we present the case of a young woman with an auricular nodular fasciitis as an example of one of the rarest sites of this tumor. The patient underwent an excision of the lesion under general anesthesia. The literature choices for treatment include complete excision, partial excision, or intralesional injection of steroids. Due to its associated local discomfort, and in order to exclude other differential diagnosis, we recommend a complete surgical excision.

**Conclusion:**

Auricular nodular fasciitis is a rare lesion. Due to its associated local discomfort, and in order to exclude other differential diagnosis, we recommend a complete surgical excision.

## Introduction

Nodular fasciitis (NF) is described as a benign reactive proliferation of myofibroblasts. The exact etiology is still unknown, but a number of hypothesis regard a preceding trauma as an etiologic factor. However, an obvious history of trauma is not always evident among patients (1,2). Due to its rapid-growing nature, a precise clinical diagnosis is difficult and the condition is frequently misdiagnosed as aggressive or malignant lesions.

Nodular fasciitis is typically seen on the extremities of adults. A head and neck lesion is most commonly seen in children, whereas the auricular lesion is rarely seen. In a study by Thompson, auricular NF accounted for 1.5% of 3930 NF cases and just 1.9% of all auricular lesions (3).

In this study, we will present the case of a young woman with an auricular nodular fasciitis. This report was approved by the Institutional Review Board of the Mashhad University of Medical Sciences.

## Case Report

A 25-year-old woman was admitted to our otorhinolaryngology clinic at Quaem Hospital at Mashhad University of Medical Sciences with a complaint of a non-tender, rapidly growing mass first observed five weeks earlier on her right auricle. There were no other accompanying symptoms.

**Fig1:**
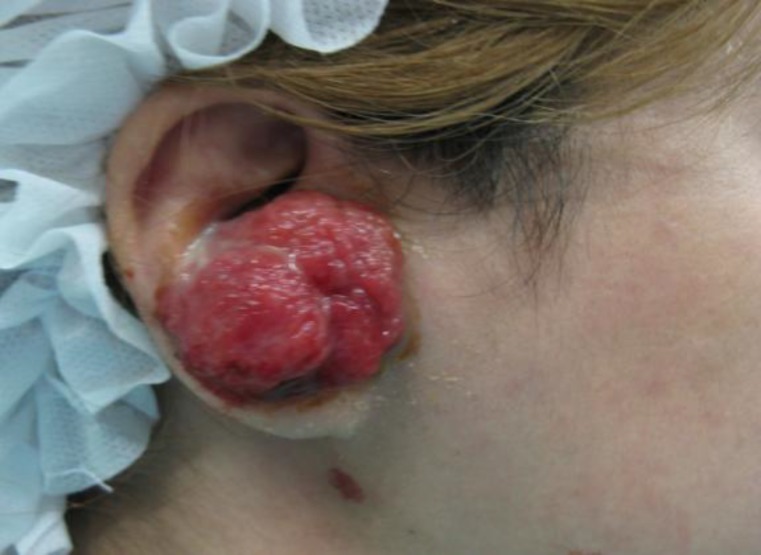
The gross appearance of nodular fasciitis lesion

On physical examination, a 2 × cm mass lesion was observed on the right auricle attached to the concha. The mass was fragile and easily became bloody by manipulation. It was not possible to examine the external auditory canal (EAC) and tympanic membrane, but the lesion appeared to be discrete from the EAC. No cervical lymphadenopathies were detected. A thorough whole-body skin examination revealed no other lesions. With a pre-diagnosis of a pyogenic granuloma, and also to rule out the malignant lesions such as sarcoma, a punch biopsy was taken. At subsequent 1-week follow up, the lesion had grown to approximately 3 × 4.5 cm (Fig1). 

**Fig 2:**
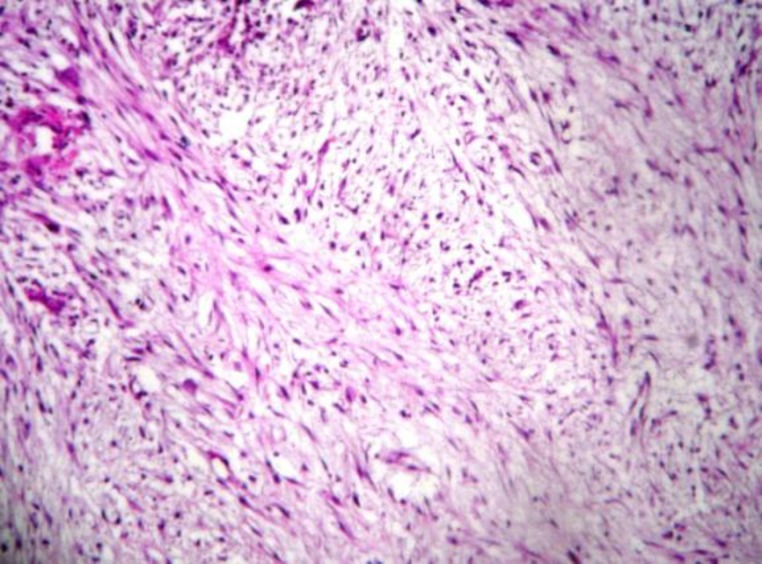
Irregular bundles of plump spindle-shaped cells and loosely arranged fibroblast in a myxoid matrix Hematoxilin-eosin staining X100 (The extravasation of red blood cells is evident).

Microscopic findings showed a proliferation of plump, mitotically active spindle-shaped cells with oval, pale-staining nuclei and short irregular bundles in a loosely mucoid matrix with extravasated red blood cells and multinucleated giant cells. An immunohisto- chemical (IHC) panel for cytokeratin (CK), S-100, epithelial membrane antigen (EMA), smooth muscle actin (SMA), and vimentin (Vim) was performed. On IHC study, CK, EMA, and S-100 were negative, and SMA and Vim were expressed, which confirms the diagnosis of NF (Fig. 2). 

According to the pathologic diagnosis of nodular fasciitis, the patient underwent an excision of the lesion under general anesthesia. The basal skin of the lesion was completely excised and the surrounding skin was primarily repaired. There was no cartilage invasion. The permanent pathologic result was also compatible with nodular fasciitis. There was no recurrence after a year.

## Discussion

Nodular fasciitis of the auricle is an uncommon lesion. It was first described by Konwaler et al in 1955 (2). In an article reviewing 50 cases of auricular NF, Thompson et al commented that this lesion is most commonly seen in young adults with a mean age of 27 years, which is similar to our patient, but is usually small in size, which differs from our case. The mean size of the lesion is reported as 1.9 cm. Other studies have reported larger lesions, also up to 10.5 cm but not usually exceeding 4 cm (4), but in our patient the lesion had grown rapidly up to 5 cm in diameter. 

In addition to its rapidly growing nature, the presence of high cellularity and considerable mitosis are the principal elements of a differential diagnosis (3). Thompson et al also observed that auricular NF is usually misdiagnosed. In their review, 75% of patients were misdiagnosed with sarcoma (3). We also considered pyogenic granuloma and sarcoma as the two most likely diagnoses before performing the biopsy.

The pathologic characteristics are consistent with other studies. Only a few spindle sarcomas grow as rapidly as nodular fasciitis, but they show histologically a greater degree of pleomorphism and a greater area of necrosis. Fibromatosis is another differential diagnosis, but fibromatosis has larger infiltrative margins with collagenous stroma and scanty inflammatory cells, which differentiates it from NF.

The treatment is also controversial. While complete excision is recommended by some authors (3,4), others have recommended partial excision (5) or intralesional injection of steroids (1). The lesion is also regarded as self limiting in some reports (1). 

## Conclusion

Auricular nodular fasciitis is a rare lesion. Due to its associated local discomfort, and in order to exclude other differential diagnosis, we recommend a complete surgical excision.
